# Supplementary material for: Establishment of a Prediction Model for Overall Survival after Stereotactic Body Radiation Therapy for Primary Non-Small Cell Lung Cancer Using Radiomics Analysis
Source: Cancers (Basel). 2022 Aug 10;14(16):3859. doi: 10.3390/cancers14163859 (PMC9405862; doi:10.3390/cancers14163859)
Supplement: Supplementary file 1 [file cancers-14-03859-s001.zip › cancers-1819309-supplementary.pdf]

Table S1. List of all 107 radiomic features.

Category	Radiomic feature
First order	10Percentile
	90Percentile
	Energy
	Entropy
	InterquartileRange
	Kurtosis
	Maximum
	MeanAbsoluteDeviation
	Mean
	Median
	Minimum
	Range
	RobustMeanAbsoluteDeviation
	RootMeanSquared
	Skewness
	TotalEnergy
	Uniformity
	Variance
Shape	Elongation
	Flatness
	LeastAxisLength
	MajorAxisLength
	Maximum2DDiameterColumn
	Maximum2DDiameterRow
	Maximum2DDiameterSlice
	Maximum3DDiameter
	MeshVolume
	MinorAxisLength
	Sphericity
	SurfaceArea
	SurfaceVolumeRatio
	VoxelVolume
Gray-level co-occurrence matrix	Autocorrelation
	ClusterProminence
	ClusterShade
	ClusterTendency
	Contrast
	Correlation
	DifferenceAverage
	DifferenceEntropy

	DifferenceVariance
	Id
	Idm
	Idmn
	Idn
	Imc1
	Imc2
	InverseVariance
	JointAverage
	JointEnergy
	JointEntropy
	MCC
	MaximumProbability
	SumAverage
	SumEntropy
	SumSquares
Gray-level size-zone matrix	GrayLevelNonUniformity
	GrayLevelNonUniformityNormalized
	GrayLevelVariance
	HighGrayLevelZoneEmphasis
	LargeAreaEmphasis
	LargeAreaHighGrayLevelEmphasis
	LargeAreaLowGrayLevelEmphasis
	LowGrayLevelZoneEmphasis
	SizeZoneNonUniformity
	SizeZoneNonUniformityNormalized
	SmallAreaEmphasis
	SmallAreaHighGrayLevelEmphasis
	SmallAreaLowGrayLevelEmphasis
	ZoneEntropy
	ZonePercentage
	ZoneVariance
Gray-level run-length matrix	GrayLevelNonUniformity
	GrayLevelNonUniformityNormalized
	GrayLevelVariance
	HighGrayLevelRunEmphasis
	LongRunEmphasis
	LongRunHighGrayLevelEmphasis
	LongRunLowGrayLevelEmphasis
	LowGrayLevelRunEmphasis
	RunEntropy
	RunLengthNonUniformity
	RunLengthNonUniformityNormalized

	RunPercentage
	RunVariance
	ShortRunEmphasis
	ShortRunHighGrayLevelEmphasis
	ShortRunLowGrayLevelEmphasis
Neighboring gray-tone difference matrix	Busyness
	Coarseness
	Complexity
	Contrast
	Strength
Gray-level dependence matrix	DependenceEntropy
	DependenceNonUniformity
	DependenceNonUniformityNormalized
	DependenceVariance
	GrayLevelNonUniformity
	GrayLevelVariance
	HighGrayLevelEmphasis
	LargeDependenceEmphasis
	LargeDependenceHighGrayLevelEmphasis
	LargeDependenceLowGrayLevelEmphasis
	LowGrayLevelEmphasis
	SmallDependenceEmphasis
	SmallDependenceHighGrayLevelEmphasis
	SmallDependenceLowGrayLevelEmphasis
